# Gender differences in the prosecution of police assault: Evidence from a natural experiment in Sweden

**DOI:** 10.1371/journal.pone.0235894

**Published:** 2020-07-22

**Authors:** Kristine Eck, Charles Crabtree

**Affiliations:** 1 Department of Peace and Conflict Research, Uppsala University, Uppsala, Sweden; 2 Tokyo Foundation for Policy Research, Tokyo, Japan; 3 Department of Government, Dartmouth College, Hanover, New Hampshire, United States of America; Rice University, UNITED STATES

## Abstract

States often seek to regulate the use of police force though citizen complaint systems. This paper examines these systems, specifically, whether patterns of bias found in other juridical contexts are mirrored in the adjudication of police assault. The analysis focuses on prosecutors as the first instance of adjudication who determine whether to move forward with investigation, effectively deciding the majority of cases. We ask whether prosecutor sex is associated with the probability that a police assault claim will be investigated. We leverage a natural experiment in Sweden where prosecutors are assigned through a modified lottery system, effectively randomizing appointment. Our findings suggest that prosecutor gender plays a role in judicial outcomes: women prosecutors are 16 percentage points more likely to investigate claims of police assault than their male counterparts. These findings have implications for scholars interested in state human rights abuses, democratic institutions, and judicial inequality.

## Introduction

Police violence is one of the most ubiquitous forms of state human rights abuse [1]. While most legal systems provide allowances for use of force in the execution of their jobs, police officers can overstep these bounds and engage in practices that violate national and international laws. To ensure that the state exercises its monopoly on violence, many democracies have instituted systems designed to discipline police violations of citizens’ rights. We know from previous research, though, that in practice justice is rarely applied blindly; ascriptive traits have often been found to correlate with judicial outcomes. This article focuses on whether police assault claims are treated equally or whether patterns of bias found in other juridical contexts are mirrored in the adjudication of police assault.

In this paper, we examine this topic in the context of prosecutors because they represent the first instance of adjudication. Most claims of abuse are determined by prosecutors when they decide whether to move forward with investigation and prosecution, making prosecutorial discretion central to the legal process. We ask whether the sex of the prosecutor is associated with the probability that a police assault claim will be investigated. Previous research on judicial equality suggests that the sex of a judge may impact on their decision [2]; we are interested in extending this line of inquiry to prosecutors. To our knowledge, no previous research exists on whether the sex of the prosecutor is associated with differential decisionmaking patterns.

We address this gap in the literature by leveraging a natural experiment from Sweden, where prosecutors are assigned to police assault cases through a modified lottery system. The randomization of prosecutorial appointment provides us with a credible identification strategy that facilitates causal inference. Beyond offering an attractive research design, Sweden also serves as an interesting case to examine this question because it is arguably a least likely case for finding any gender effects. As one of the most gender-balanced countries in the world [3], we expect that if gendered effects exist there, these findings would travel elsewhere.

The Sweden case is also advantageous for practical reasons. The Swedish law on public access to information allows us to obtain these data; the same law also facilitates citizen reporting of perceived police assault because it ensures transparency and accountability within the legal system [1]. The threshold for filing a claim is low: a citizen need only state their desire to do so to an agent of the police and a file is immediately opened and sent to an independent special prosecutor. There is little reason to fear recrimination for filing a complaint, nor is much (or any) remuneration awarded if the police officer is found guilty, so complaints are likely to be genuine expressions of grievance. While Sweden has a well-deserved reputation for respecting human rights, like in most countries, accusations of violations by individual police officers do occur on a regular basis. In this sense, the Swedish government is in a position typical of many democratic governments which struggle to find ways to contain their agents.

Our findings suggest that prosecutor gender plays a role in judicial outcomes: the probability of a police assault claim being investigated is 16 percentage points higher when the case is assigned to a female prosecutor. From the perspective of those who advocate for human rights and the promotion of democracy, this is problematic. For citizens who believe themselves to be victims of abuse, it is likely to seem unjust that their claims are less likely to be investigated if assigned to male prosecutors. Because of the process by which cases are assigned to prosecutors, we can be confident that this finding is not driven by the quality and characteristics of the claims or claimants themselves, nor those of the officers who are accused. While we are able to identify the causal effect of a police complaint being assigned to a female prosecutor, our research design and data do not allow us to adjudicate between the possible mechanisms that might drive this effect. We therefore conclude the article by offering some suggestions for future research in unpacking the reasons behind why men are less likely to investigate police assault claims.

## Literature review

The literature on equality under the law largely focuses on whether ascriptive attributes of judges are associated with their decisions, focusing on the sex or racial characteristics of judges in the Unites States. The standard methodological approach is to regress the judge’s vote on the ascriptive attribute of interest and a fairly uniform set of covariates relating to other attributes of the judge and/or case, although researchers are increasingly moving beyond this standard analysis of observational data by using matching and other techniques designed to improve inference [2, 4–8].

Within this literature, the findings for the effect of sex on judging are indeterminate. Our focus here is on individual effects, but the field has also examined group effects, i.e. whether the presence of a woman judge on a court impacts on the panel’s decisions [2, 9]. Many studies find no difference between female and male trial judges across a range of issue areas [2, 10–13]. This includes issues traditionally considered to be of particular interest to women, such as gender discrimination, sexual harassment, abortion rights and maternity rights, custody battles, and equal pay than their male counterparts [14–16]. Other studies have found that women judges are more likely to rule in favor of the defendant in Fourth Amendment search and seizure cases [9], to vote liberally in death penalty cases [13], to harshly sentence criminal defendants [17–19], to rule in favor of the plaintiff in sex discrimination in employment cases [2, 13, 20, 21], and to make “pro-women” decisions [22]. A handful of jury studies have found some evidence of gender effects. One historical pre-post design shows that the inclusion of women on juries impacted on conviction rates in female-salient cases [23], while another found gender effects conditional on race in North Carolina [4]. Using data from several counties in Florida, Hoekstra and Street [5] find that own-gender juries result in lower conviction rates for drug charges, but not other charges.

Scholars have noted an overwhelming focus on judges at the expense of other phases of the adjudication process. In particular, the role of the prosecutor has received little attention, which is problematic given that they determine which cases to pursue. The research on prosecutors has focused on the attributes of the cases and claimants in explaining which cases are pursued and their outcomes, using both observational and quasi-experimental designs [7, 8, 24–34] (Hepburn, 1996). A burgeoning literature has also begun to examine the relationship between *prosecutor* attributes and behavior. Metcalfe [35] examines the relationships between prosecutors and defense attorneys and prosecutors and judges in explaining plea bargains. Arora (2018) exploits a quasi-experimental design to identify that Republican prosecutorial offices sentence defendants to longer sentences than Democratic offices. In a design similar to ours, Sloane [6] leverages as-if random assignment of prosecutors in New York country, finding that prosecutor race influences conviction rates, conditional on defendant race. None of these studies have examined the gender characteristics of the prosecutor. Given the wide ambit under which prosecutors operate, the lack of research on prosecutorial discretion is a central lacuna to contemporary research on courts and sentencing.

The lack of research on this topic is likely attributable to data availability problems, as data on the characteristics of prosecutors are less likely to be public compared to judges.

### Prosecutorial bias in police assault

Our study of prosecutorial biases focuses on an issue area which has received little attention in the literature: the adjudication of police assualt or misconduct complaints. Police assault poses an institutional challenge because it is agents of the state who are both under investigation and who are the first instance for citizen recompense. We examine whether prosecutor gender impacts on how police assault complaints are processed in the criminal justice system. Because the use of violence in policing is a core facet of state respect for human rights, we view any systematic bias in how claims are treated to be problematic for the exercise of equality under the law.

Media coverage of police brutality in the United States has propelled this issue to the forefront of national conversations about what is appropriate violence. The United States is not the only country to struggle with determining and enforcing the boundaries of police behavior. Though reliable global statistics on the phenomenon do not exist, human rights reports make it clear that excessive violence by the police is widespread globally. This should not be surprising: police regularly put themselves in potentially dangerous situations and work under conditions of heightened threat. Their mandate to ensure order combined with permission to use force gives them considerable leeway in the application of violence. Given this intersection of threat perception and power to use violence, it occurs with varying degrees of regularity that individual police officers use more force than strictly needed in encounters with the public.

Most democratic countries have some sort of institutional mechanism for addressing police assault complaints but these systems vary widely. In the United States, complaints can be adjudicated through both criminal and civil law procedures. In the criminal law system, investigations are primarily conducted by internal police commissions or district attorneys. Both approaches have been criticized for a lack of independence, since district attorneys are dependent on police cooperation to bring cases to trial, making them reliant on upholding a good working relationship. Police departments facing citizen outcry have occasionally created independent commissions, such as the Independent Commission of the Los Angeles Police Department in the wake of the Rodney King case. At the federal level, the Department of Justice has used legal provisions to influence reform in some jurisdictions, but no national-level oversight system exists. Indeed, the federal government is reliant on states to collect and report data on police homicides to the Bureau of Justice Statistics, and a majority of states monitor police homicides through local media reports rather than internal documentation [36]. For point of contrast, Canada has created an Office of the Police Complaint Commissioner (OPCC), an independent office under the legislature staffed by civilians.

Governments can thus generate accountability by creating institutions that circumscribe the use of force by police. But equally important is the question of how these institutions function. We are aware of one study on this topic which focuses on 170 cases of police brutality adjudicated by U.S. state supreme court judges [37]. It finds that women justices are more likely to rule in favor of the victim. Methodologically, the study does not represent a random selection from the population of police brutality claims, but those which were actually brought to trial and advanced to the state supreme court, indicating that there are likely to be selection effects in which cases were adjudicated. Nonetheless, this study suggests that there may be gender effects in the adjudication of police brutality cases. Our focus, however, is on the *prosecutor* in this process because it is the prosecutor who makes the first, and critical, decision whether there is sufficient merit to a claim to warrant opening an investigation. This decision is essential because a majority of cases are weeded out in this stage.

### Theory

In theorizing about the relationship between prosecutor gender and the police assault claims that they adjudicate, one starting point is the issue of police assault itself. It is possible that on average women exhibit stronger commitments to human rights values or to processes of justice than do men. Previous research has found that women judges are more likely to support claims in cases related to freedoms protected by the Bill of Rights and by state and federal law [15]. More broadly, men are more likely to support military programs and punitive policies while women are more likely to express support for social programs and equal rights [38–40]. Men’s higher levels of support for or tolerance of violence in a variety of forms, including police violence, is well established [37, 41].

Along the same lines, sex could also capture ideological differences regarding what dispensations for violence should be allowed government agents in the name of law and order imperatives. Findings from social dominance theory in psychology has found that men are more likely to exhibit social dominance traits [42] and that these traits are associated with a belief that harming people is legitimate. Researchers have sought to explain gender differences in militaristic attitudes as functioning via male inclinations to social dominance; many of the policies and ideologies that men express greater support for, including militarism, coercion, racism and patriotism, have to do with the domination of one social group over another. High levels of social dominance in this context could imply belonging to an in-group composed of agents of law and order which views complainants as belonging to an out-group of criminals or social troublemakers.

The possibility of a gender effect may also be unrelated to the nature of the cases being adjudicated. There is a cross-disciplinary literature that shows that women exhibit greater diligence in performing duties. Studies of personality traits show that women exhibit higher levels of dutifulness than men [42], a pattern which is replicated in schools [43] and the work force [58, 59]. This performance drive may be due to intrinsic standards, but it may also be driven by a perceived need to work harder in order to achieve career success. In addition to direct economic benefits, high performance could be driven by a desire for prestige or to improve future promotion opportunities, and high case disposition rates are likely to enhance a prosecutor’s reputation.

Reputational concerns may also be driving the women prosecutors’ increased propensity to investigate cases. Women prosecutors may feel a need to appear tough on crime in order to counteract gender normative perceptions of women as weak. The fact that the possible crime in question is being committed by a police officer, however, complicates this story somewhat: it is unclear whether would one appear more strong by being seen to hold police accountable for their abuses, or by siding with the forces of law and order and allowing them leeway in their application of violence. While reputational considerations are likely to be complex, it is plausible that they influence prosecutors execution of their job.

This leads us to hypothesize that women prosecutors are more likely to investigate police assault claims:

*H1a: Women prosecutors are more likely to investigate complaints of police assault*.

At the same time, a countervailing school of thought stresses the importance of professional training which is designed to harmonize decision-making and ensuring equality in the execution of legal duties. This organizational model suggests that the common elements of legal training, shared norms, and identical constraints imposed by organizational rules should negate personality or background differences across individuals [19, 44]. This approach rejects ideas of subjectivity in the judicial system and promotes a belief that the ideal of impartial and objective decision-making can be achieved. This approach leads us to an alternative hypothesis which posits that there will be no prosecutorial gender effect:

*H1b: There is no effect of prosecutor sex on the likelihood of investigating police assault complaints*.

### Case selection

To investigate the relationship between prosecutor sex and the adjudication of police assault claims, we turn to the case of Sweden. We do so primarily because it provides us a natural experiment through which we can identify the effect of a case being assigned to a female prosecutor. But there are other reasons to examine police assault in this context. First, Sweden is one of the most gender equal countries in the world [3], where norms of equality have been actively promoted by the state. While Sweden has not yet achieved complete equality in any dimension (political, economic, or social), its position at the forefront makes it a least likely case to find differential patterns of behavior between men and women. The Swedish system strongly emphasizes equality under the law [45]. Thus, if we find an effect in Sweden, we feel confident in speculating about its generalizability to less gender equal contexts.

Second, the Swedish state actively promotes human rights and exhibits a high level of transparency regarding its own behavior (and failings) in this regard [1]. Norms of non-violence are deeply entrenched in Sweden and we would therefore expect that claims of violations by the state would be addressed with an aim to rectifying the problem rather than covering it up.

Third, the complaints system in Sweden is straightforward and easily accessible for the average citizen. The threshold for filing a complaint is low: a claimant need only state to the police that they would like to do so and a complaint is immediately filed on their behalf. The only cost that is incurred is the time necessary for the party to make a statement. There is little or no monetary recompense associated with complaints; even if the police officer is ultimately convicted of assault, damages paid to the victim are typically small (i.e. hundreds of US dollars). To that end, it is unlikely that citizens make false claims with the incentive of monetary gain.

Despite its relatively strong reputation for respecting human rights, Swedish agents of the state do engage in excessive violence, as the thousands of police assault complaints that compose our empirical material illustrate.

We obtained records of prosecutor decisions for all police assault complaints filed between 2013–2016. Complaints are most commonly made in the context of everyday arrests or police interventions. These complaints are directed to a body called the Separate Public Prosecution Office (*Särskilda åklagarkammaren*, hereafter SÅK*)*. While it is part of the Swedish Prosecution Authority administratively, it answers directly to the Prosecutor-General because it deals exclusively with complaints registered against other agents of the state (police, judges, prosecutors, parliamentarians, etc.) and is therefore removed from other police and prosecutorial organs. It is a centralized, national-level body. Complaints sent to SÅK are distributed to special prosecutors, who then determine whether there is reason to initiate an investigation. We discuss the assignment process in the next section.

If the prosecutor initiates an investigation, they typically make requests to the police to obtain further information, such as statements, video surveillance footage, hospital records, etc. On this basis, the prosecutor then decides whether to close the investigation or whether to proceed to charges. Summary reports for 2012–2014 suggest that less than 2% of all misconduct complaints result in punishment (either fines or criminal charges) [46]. Our data for 2013–2016 suggest that these rates may be even lower for assault charges: only 30 of the almost 3,300 assault complaints resulted in censure.

## Materials and methods

Our research design exploits a natural experiment in prosecutorial case assignment. Before we describe our identification strategy, we first provide some context about how Sweden investigates complaints against the police. There are two prosecutorial tracks for allegations of police assault. After a complaint is registered, the police make an initial determination about the merits of the complaint. If they believe that the complaint is unlikely to have legal standing, they send it to a special prosecutor assigned to handle these “fast track” complaints. These claims are evaluated according to the same legal criteria as all others. They are typically dismissed, though, because they fail to meet the legal criteria for assault. They might be missing necessary documentation, for example, which could happen if the complainant fails to provide a statement. They also might include claims that misunderstand the nature of “assault,” such as allegations by individuals that they were assaulted because they were placed in handcuffs. Because we know the prosecutor assigned to the fast track, we are able to omit these extraneous complaints, which compose approximately 29% (958) of the total population of cases from 2013–2016 (3,291). In 2018, the Parliamentary Ombudsman conducted an independent investigation of the Special Prosecutors Authority which, with few exceptions, supported the decisions taken by the fast track prosecutor to not investigate [47].

The remaining cases go through the second, “normal” track. These cases are allotted to special prosecutors by the Director of Public Prosecution, who is the head of the Special Public Prosecution Office. In theory, he assigns these cases to prosecutors on a rotating basis. In this way, the assignment mechanism here is similar to that described in Loewen et al [48]. This assignment system is complicated, however, by the fact that prosecutors may not work full time or may have other assigned tasks aside from investigating police assault. As a consequence, the Director assigns cases in a modified lottery process that is based on a rotation schedule but also takes into account the current workload of the available prosecutors. Crucially for our research design, he does not read the case prior to assignment. This means that he does not take into consideration the merits of cases or any other characteristics while assigning them, beyond the fact that they have been assigned to the “normal” track. Just as importantly, prosecutors cannot select into certain types of cases; they must work with those they are dealt. The prosecutors are a homogeneous pool with respect to their careers; to work as a special prosecutor requires extensive experience and a track-record of integrity. As a result, the prosecutors are all in roughly the same high age cohort, with an average age of 58, and have extensive experience working in the national judiciary [49, 50]. This homogeneity helps us to isolate the gender effect and alleviate concerns that gender might be proxying age or professional experience. The Director of Public Prosecution has clarified for us that there are no difference between male and female prosecutors with regard to age, experience, or workload [50]. Prosecutors are independent from direction by their superiors in making case-level decisions [45]. Note also that all Swedish prosecutors are selected meritocratically and are seen as civil servants; they are not chosen for political ideological reasons nor are they supposed to reflect the views of the public [45].

This assignment process provides us with a natural experiment in which cases are randomly assigned to prosecutors of different genders. This means that the characteristics of the case should be exogenous to the gender of the prosecutors, which removes from consideration the typical selection problem in studies like this, where prosecutors usually pick the cases they investigate. Like all natural experiments, though, ours must rely on some identifying assumptions [51]. The key assumption is that prosecutorial availability (i.e. case load) is not correlated with (i.e. is exogenous to) the gender of prosecutors **and** the outcome of the case investigation. Our conversations with the Director of Public Prosecution suggests that this assumption is reasonable.

Our data set represents almost the entire population of non-fast-track complaints filed from 2013 to 2016. All data files used in our analyses are available from github.com/cdcrabtree/brutality, along with a data description and the code necessary to replicate our analyses. From the non-‘fast track’ pool of complaints we only dropped those that were prosecuted (*n* = 29). We did this to ease analysis. Our results are substantively the same if we leave that small subset of observations in the data. Our primary interest is in assessing the causal effect of being assigned a female prosecutor on complaint resolution. To make this inference, we do not need a representative sample of complaints across time, but a research design that can provide us with internally valid claims. As Crabtree and Fariss [52] note, it is important to first verify the internal validity of theoretical claims before assessing the degree to which those claims extend to other samples. We think that a fruitful avenue for future work would be to test how our findings across time and across space.

We extract several data from the complaints. An example complaint is included in the online appendix. First, we code the name of prosecutor that was assigned to the complaint. From this, we create our treatment indicator, Female, which is coded ‘1’ if a case is assigned to a female prosecutor and ‘0’ otherwise. In order to create this measure, we assume that traditionally female given names are associated with sexual attributes. We acknowledge the cisgender assumptions built into this assumption. All of the prosecutors names are common Swedish given names and were not ambiguous with regard to sex; for example, ‘Anders’ is a given name traditionally associated with men in Sweden, while ‘Anna’ is traditionally associated with women. These are typical examples drawn from the data; we are therefore confident that we have correctly assigned sex to the prosecutors.

Second, we capture the outcome of the complaint, Investigation, which is coded ‘1’ if a prosecutor decides that a complaint should be investigated and ‘0’ otherwise. This is our dependent variable. Third, we code the year that the complaint was filed. Fourth, we code whether the complaint was filed during the typical summer vacation period in Sweden. We collect these data to rule out that any patterns we observe are driven by or limited to specific periods within our data set. Taken together, our collected measure represents nearly the entirety of data that can be extracted from the reports provided to us.

**Fig 1:**
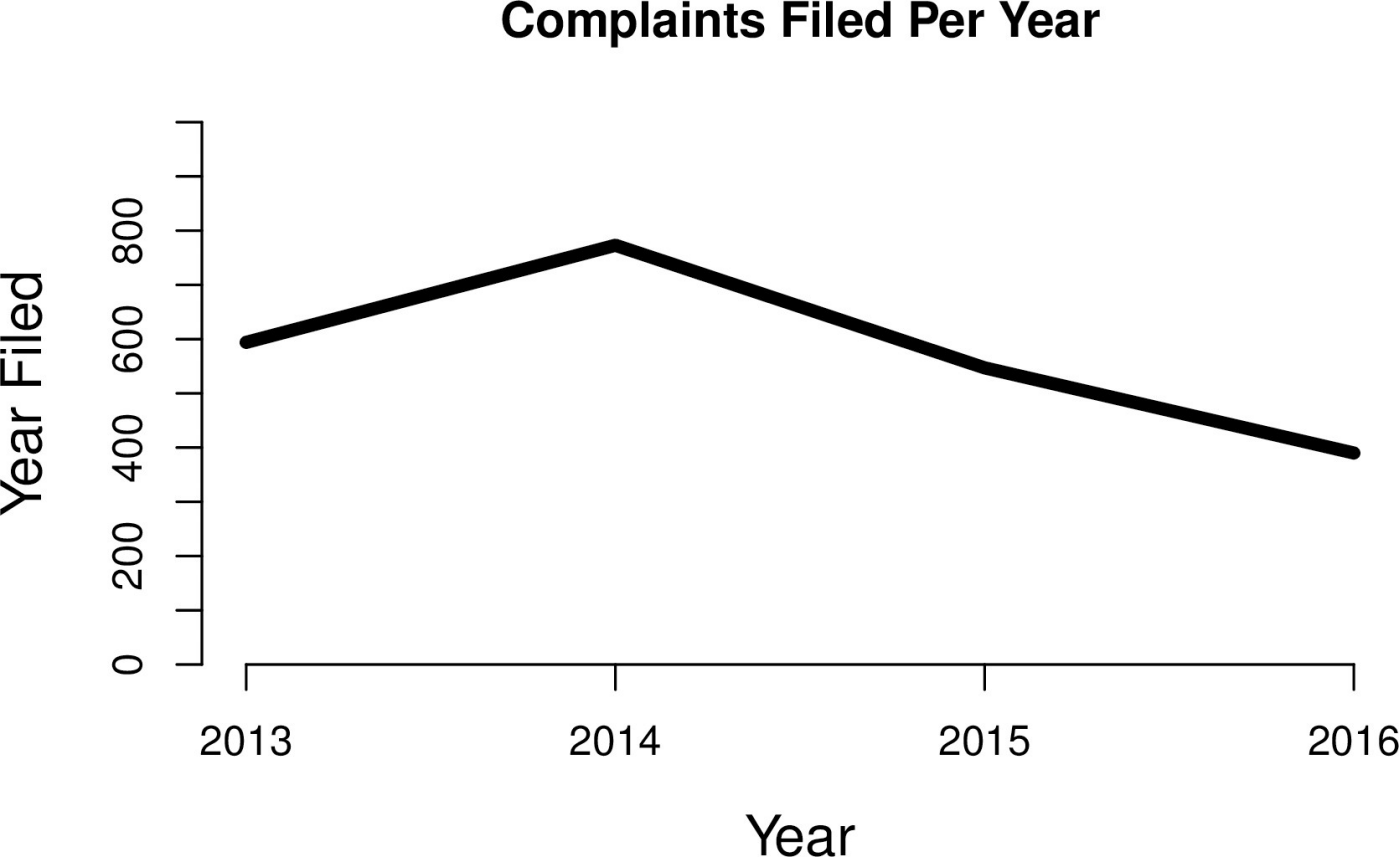
Descriptive data on complaints.

Fig 1 displays the number of complaints filed per year. It shows that the number of complaints has decreased over time. As we discuss later, we take account of this temporal heterogeneity by including year fixed effects in some of our model specifications.

As a reminder, each one of these complaints was assigned via a lottery system to an individual prosecutor. While the number of prosecutors available to handle these cases varies across years, nineteen different prosecutors handled the cases in our sample. For the purposes of our research design, these prosecutors can be considered as different treatments assigned to cases, our unit of interest (*n* = 2,304). Eight of the prosecutors are female and eleven are male. The number of male prosecutors assigned cases stays about the same throughout our panel, but the number of female prosecutors increases from four in 2013 to seven in 2016, which may reflect an attempt by the agency to increase gender balance in staffing. As we would expect based on the gender imbalance in the prosecutorial pool, SÅK assigned more cases to male prosecutors (1,267) than female prosecutors (1,037).

One way of thinking about this gender imbalance is that the probability of being assigned to the treatment condition (i.e. female prosecutor) was lower than the probability of being assigned to the control condition (i.e. male prosecutor). This is not problematic for the inferences we draw, though, since assignment to treatment is still random. One potential issue, though, might be that our results are limited to the specific prosecutors (i.e. treatments) that were assigned cases. A productive area of future research would be to test the scope conditions of our findings by leveraging similar lottery systems in other contexts.

After being assigned a complaint, a prosecutor needs to determine whether the case should be investigated or not. The most common outcome is that a case is investigated. About 32% of cases were not investigated (*n* = 748), while 68% of cases were investigated (*n* = 1,556).

To analyze the effect of prosecutor gender on case outcomes, we estimate a series of linear probability, logit, and probit models. We describe those models and their results in the next section.

## Results

To determine whether the outcome of the case vary based on whether it was assigned to a female prosecutor, we first examine whether the number of complaints investigated varies based on the gender of the prosecutor. In our between-case data, we find that female prosecutors investigate almost 78% of complaints, while male prosecutors only investigate about 60%. This difference implies that gender matters and we next turn to a more thorough analysis of this claim.

To see if this difference in prosecution rates between males and females is statistically and substantively significant, we conduct a series of statistical analyses using R. In all our tests, we use a threshold for significance (i.e. alpha level) of 0.05. As one might expect from the figure above, a Chi-squared test shows that this difference in prosecutorial investigation patterns is statistically significant (*p* < 0.001). One possible concern might be that our sample is not large enough and so Fisher’s exact test should be preferred. We obtain similar results (i.e. *p* < 0.001) if we use that test or Barnard’s test instead. One might also be concerned that this pattern in the pooled data is driven by statistical quirks in certain years, but the general pattern is remarkably stable across time–female prosecutors are more likely to launch official investigations into citizen complaints across all years. Taken together, the data and test suggest that female prosecutorial effect on case investigation seems to be both positive and substantively meaningful.

While this test indicates that the outcomes of complaints are different depending on whether they have been assigned a female or male prosecutor, it does not allow us to assess the substantive effect of prosecutor gender. To investigate this, we estimate a series of ordinary least square regression models. More specifically, since our dependent variable for these models, Investigation, is a binary indicate we estimate a set of linear probability models (LPM). While our outcome measure, is binary, we use LPMs because the results are easy to interpret and because coefficient estimates are unbiased if the model is specified correctly. Our model is specified correctly since we only include dummy variables [53]. To ensure that our results are not model dependent, we also estimate logit and probit models. The results from these models, which we report in the online appendix, are substantively the same.

The primary independent variable is our treatment indicator, Female. We use HC2 robust standard errors to account for heterogeneity in the error term. We obtain similar results if we use classic standard errors. One might be concerned that we should use clustered standard errors. The literature, however, is unclear about whether cluster standard errors should be used when randomization occurs at the unit level [54]. Another issue here is that the literature is also unclear about when the number of clusters is sufficiently large to justify the asymptotic assumptions underlying traditional cluster-robust standard errors. Acknowledging the potential problems with using cluster standard errors given the randomization scheme employed by the lottery, we address the second issue by using bootstrap clustered standard errors. We use several variants of these errors as robustness checks, clustering on the year, prosecutor, and (non-)vacation period. Using different variance-covariance matrices does not affect our results.

Our model specification is below in Eq (1).

$$I{\mathrm{NVESTIGATION}}_{i}=\beta_{0}+\beta_{1}\;F{\mathrm{EMALE}}_{i}$$ (1)

**Fig 2:**
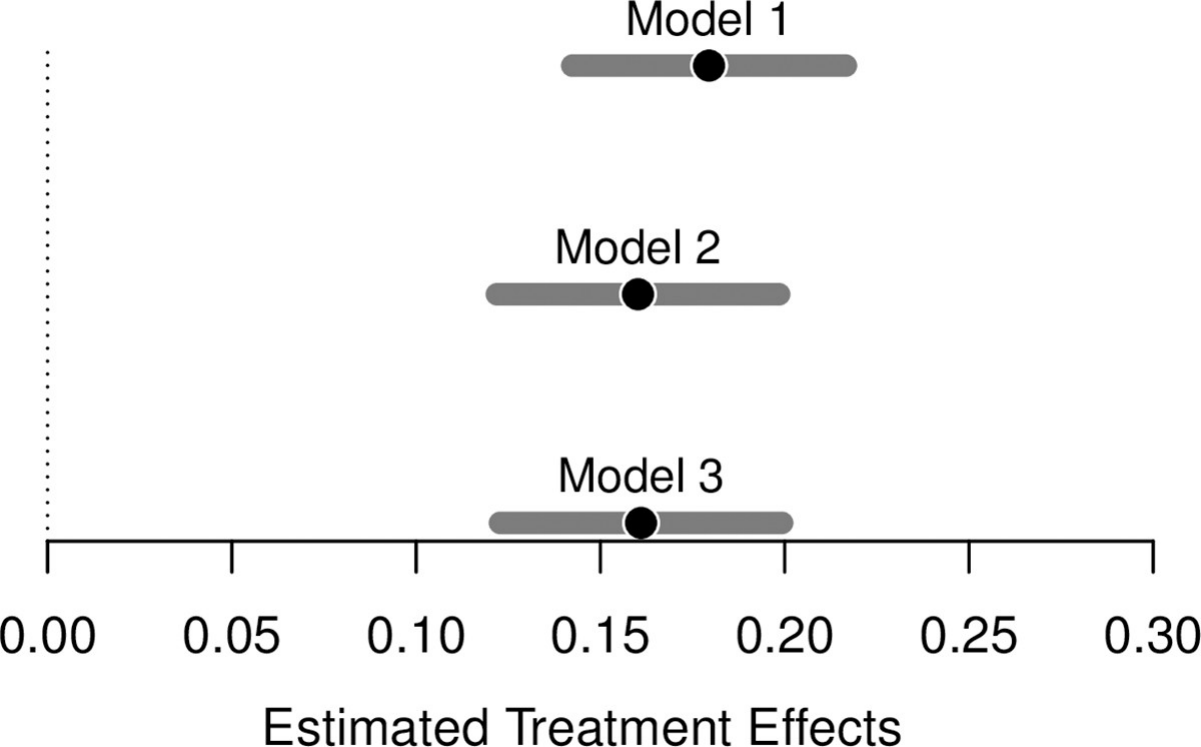
LPM model results. Fig 2 shows the results from several LPM models. The figure plots the estimated coefficients (black points) from the model along with 95 percent confidence intervals (gray bars). The reference category across models is ‘male prosecutor’.

Fig 2 shows the results for this model and several other models. Tabular results can be found in the online appendix. The figure plots the estimated coefficients (black points) from the model along with 95 percent confidence intervals (gray bars). The reference category for Model 1 (and other models) is ‘male prosecutor’. As can be seen clearly in the plot, the estimated effect of Female from Model 1 is positive and statistically significant. This is in line with our theoretical expectations. The effect is also substantively important. It suggests that if a complaint is assigned a female prosecutor, the probability of the complaint being investigated increases by about *18* percentage points.

One concern here might be that we have not accounted for different temporal trends in the data. While this should not be an issue since the ‘female prosecutor’ treatment has been randomly assigned, we re-estimate Eq 1 but now include year fixed effects (**X**_i_). This means that in Model 2 we are now identifying the causal effect of our treatment within years. The estimated coefficient from this model is presented in Fig 2. Another reason to include year fixed effects might be if we think that temporal trends are predictive of the outcome. In this case, including dummies for years in the model would increase the precision of our estimates. As can be seen in Fig 2, though, the estimated effect of Female is already very precise.

Comparing this effect to the one from Model 1, we see that accounting for time shocks does not change the statistical significance of Female. The substantive significance is also virtually unchanged. If a case is assigned to a female prosecutor the probability of it being investigated is around 16 percentage points higher. Again, police officers appear to have a much easier time if a complaint against them is assigned to a male prosecutor.

As an additional check that our findings are not being influenced by some time-specific factor, we re-estimate Eq 1 with year fixed effects but also a dummy variable for whether a case was opened during the yearly vacation period (**X**_i_ + β2 vacationi). Consequently, in Model 3 we are identifying the causal effect of our treatment within years and within (non-)vacation periods. Since treatment was randomly assigned, we would not suppose the results of this model to be different than those from Model 1 and Model 2. In line with this expectation, we find that the estimated coefficient from Model 3, presented in Fig 2, is in line with prior findings. Once again, we see that the effect of Female on complaint investigation is substantively and statistically significant.

To further address potential concerns that our findings are driven by time-specific factors, we transform our panel data into four separate cross-sections and re-estimate Model 3 with these yearly slices of our data. Since we are only using data for individual years, we must omit year fixed effects, the inclusion of which would not make sense theoretically or statistically. The results from these models, presented in the online appendix, are strikingly similar to the results presented in Fig 2. This provides strong evidence that our results are not driven by or specific to certain time periods in our data.

We conduct several additional robustness checks. First, we estimate a series of logit and probit models that mirror Models 1 through 3, finding substantive similar results; the results from these models can be found in the online appendix. Second, we address the possible objection that our results might be driven by the behavior of a small number of prosecutors. To deal with this, we use a jackknife procedure where we iteratively drop the cases associated with one of the prosecutors in our data and re-estimate our final LPM model. We obtain similar results using this procedure. Our third robustness check addresses the potential concern that the treatment effect we observe might seem too large or too precise and therefore be a ‘statistical fluke’. In order to see if that is the case, we conduct a Studentized permutation test [55]. The test allows us to compare the t-statistics from our LPM model with the “average treatment effect … under random reassignments of treatment that follow the same randomization scheme as the actual experiment” ([55], 117). We simulate 5,000 random reassignments and calculate *p*-values. Each *p*-value returned from this test represents the fraction of test statistics strictly greater than the test statistic in our sample [56]. The results from this test are very similar to those reported above. It makes sense that the permutation test would corroborate our LPM results since we have a relatively large sample and use robust standard errors [56]. A fourth possible robustness test would be to account for any time-invariant differences across prosecutors by including prosecutor fixed effects in our model. While this would be a good idea, in theory, our primary variable of interest–prosecutor gender–does not vary within prosecutors over time or across cases. As a result, if we include prosecutor fixed effects then the effect of prosecutor gender would be unidentifiable.

Taken together, the results of our empirical analyses provide suggestive evidence that female prosecutors treat police complaints differently than male prosecutors. Across our model specifications, we find that a complaint being assigned a female prosecutor increases the probability that the complaint will be investigated by approximately 16 percentage points. While we can only speculate as to the causal mechanism that drives this effect, we think that the effect is compelling, and highlights the need for additional work on the role or prosecutorial gender in judicial outcomes. One particularly promising area of future research would be to rule out the possibility that common characteristics shared by female (of male) prosecutors–such as education, personal experiences, or partisanship, might drive the observed difference between prosecutors of different genders.

## Discussion

The results show that women prosecutors are more likely than men to investigate claims of police officers assault but our data and research design do not provide us with a clear explanation as to *why* this result obtains. In our theoretical argument, we presented a number of plausible stories which could explain the finding, including differential commitment to human rights, professional diligence, career advancement, and reputational concerns. The data do not allow is to adjudicate between these (or other) possible theoretical stories, but our intuition is that the result may be a combination of several possible mechanisms. These findings indicate that the question of gender differences in prosecutorial behavior warrants more research; in particular we think a good starting point would be to explore adjudication behavior across other areas of criminal law in order to isolate whether there is a particular effect for police assault complaints or whether this is a more generalized phenomenon.

We think it also important to call attention to the fact that we have framed these explanations as if women’s behavior is exceptional and needs therefore to be explained. We do so because this is largely how the literature discusses these issues. But we think it equally appropriate to ask not why women are more likely to investigate police assault complaints but why men are less likely. Women special prosecutors are arguably just doing their jobs and we should perhaps instead explain the findings in terms of why men are less diligent, less interested in career advancement, less concerned about their reputations, or less committed to the adherence of human rights norms.

These results should be a cause for concern for individuals who believe they have been the victims of police assault, and they should hope that their complaint is assigned to a woman special prosecutor in order for it to have a better chance of being investigated. That any factor aside from the legal merits of the complaint itself should play into the adjudication process, and that it should do so systematically, is indicative of judicial inequality. These findings should be troubling for Sweden, which has sought to create a transparent and easily accessible system for citizen feedback on state abuses in order to prevent future instances. Beyond Sweden, these findings should be troubling for all states which seek to ensure equal justice since they suggest that even in a highly gender equal context, differential patterns of behavior persist. They also suggest that the role of the prosecutor warrants much more attention, given their central role in determining which cases shall move into the court system.

A cynical response to these findings might be that these differential rates of investigation do not really matter given how few investigations ultimately move to the prosecution stage. But responsiveness to citizen feedback is important to the state’s ability to legitimize its monopoly of violence. Institutions of police misconduct are designed to identify and constrain abuses, and their social value is arguably contingent on people believing that their complaints are investigated impartially and with equal care.

## Conclusions

The findings here indicate that women special prosecutors are around 16 percentage points more likely to investigate police assault in Sweden than their male counterparts. On the basis of our research design, we can rule out that the attributes of the complainant, the police officer, or the situation itself could confound these results. These findings have important implications for scholars interested in state human rights abuses, scholars of democracy interested in the functioning of institutions, and scholars of the judiciary interested in differential patterns of adjudication associated with ascriptive traits.

Police violence is largely ubiquitous across the globe. It differs in extent and scale, but it occurs in most countries most years. Most democratic states seek to design systems of citizen feedback and agent accountability in order prevent further abuses. But just as important as their design is how they function. Are they working as intended; are they effective at enforcing the laws and rules adopted to restrict police violence? Or are they characterized by their own pathologies? Our results indicate that there may be greater scope for biases to introduce unequal justice than intended.

Some potential policy implications spring from our results, although these are speculative. Our study would not have been possible with a randomized system of case assignment for investigating complaints about police misconduct. For such systems to be useful for drawing causal inference, cases should be *assigned*; there should be no opportunity for those in adjudicative roles to select themselves into cases. In the U.S. and other countries there are opportunities for judges to select cases with the motivation that they are specialists or have other relevant expertise. Previous research has demonstrated that this can lead to the appearance of bias [57] and raises questions of the legitimacy of the judicial process. Likewise, to maximize inferential leverage, case and prosecutor assignment should be *randomized*. Random assignment not only facilitates researchers’ ability to draw inferential conclusions, it also protects the system from abuse. Systems of true random assignment, however, come with a trade-off in efficiency, since they do not allow for specialization.

The findings also suggest that adjudication processes should be evaluated to detect whether the judicial system is operating as intended, or whether biases are prejudicing the outcomes of cases. Monitoring and measuring the functioning of state institutions—particularly security agents—is essential to addressing citizen perceptions in many contexts that law enforcement and adjudication are beset with inequity; these concerns are foundational for social movements and contentious politics in numerous countries. There is a clear imperative for governments to prioritize evaluation systems of their agents in order to ensure the provision of equal treatment under the law.

## References

[1] Eck K., & Fariss C.J. (2018). Ill-treatment and torture in Sweden: A critique of cross-Case comparisons. *Human Rights Quarterly*, 40, 591–604.

[2] Boyd C. L., Epstein L., & Martin A. D. (2010). Untangling the causal effects of sex on judging. *American Journal of Political Science*, 54(2), 389–411.

[3] World Economic Forum. (2017). *The global gender gap report* Available at: http://www3.weforum.org/docs/WEF_GGGR_2017.pdf Accessed 2017-11-13.

[4] Flanagan Francis X. (20918). Race, gender, and juries: Evidence from North Carolina. *The Journal of Law and Economics*, 61(2), 189–214.

[5] Hoekstra Mark, and Street Brittany. (2018). The effect of own-gender juries on conviction rates. No. w25013. National Bureau of Economic Research.

[6] Sloane, CarlyWill. (2019). Racial bias by prosecutors: Evidence from random assignment. Working paper, Texas A&M. Available at: https://github.com/carlywillsloan/Prosecutors/blob/master/191228_sloan_jmp.pdf. Accessed 2020-01-16.

[7] Tuttle, Cody. (2019). Racial disparities in federal sentencing: Evidence from drug mandatory minimums. SSRN, https://papers.ssrn.com/sol3/papers.cfm?abstract_id=3080463. Accessed 2020-01-16.

[8] Yang Crystal S. (2016). Resource constraints and the criminal justice system: Evidence from judicial vacancies. *American Economic Journal*: *Economic Policy*, 8(4), 289–332.

[9] McCall M., & McCall M. A. (2007). How far does the gender gap extend? Decision making on state Supreme Courts in Fourth Amendment cases, 1980–2000. *The Social Science Journal*, 44(1), 67–82.

[10] Ashenfelter O., Eisenberg T. & Schwab S. J. (1995). Politics and the judiciary: The influence of judicial background on case outcomes. *Journal of Legal Studies*, 24, 257–282.

[11] Davis S. (1991). The Supreme Court: Rehnquist’s or Reagan’s? *Western Political Quarterly*, 44(1), 87–99.

[12] Sisk G. C., Heise M. & Morriss M. P. (1998). Charting the influences on the judicial mind: An empirical study of judicial reasoning. *New York Law Review*, 73, 1377–500.

[13] Songer D. R., Davis S. & Haire S. (1994). A Reappraisal of diversification in the Federal Courts: Gender effects in the Court of Appeals. *Journal of Politics*, 56(2), 425–439.

[14] Kulik C. T., Perry E.L. & Pepper M.B. (2003). Here comes the judge: The influence of judge personal characteristics on federal sexual harassment case outcomes. *Law and Human Behavior*, 27, 69–86. 10.1023/a:1021678912133 12647468

[15] Segal J. A. (2000). Representative decision making on the Federal bench: Clinton’s District Court appointees. *Political Research Quarterly*, 53(1), 137–150.

[16] Walker T. G., & Barrow D. J. (1985). The diversification of the Federal bench: Policy and process ramifications. *The Journal of Politics*, 47(2), 596–617.

[17] Collins P. M. Jr., Manning K.L., & Carp R.A. (2010). Gender, critical mass, and judicial decision making. Law & Po*licy*, 32, 260–281.

[18] Spohn C. (1990). Decision making in sexual assault cases: Do black and female judges make a difference? Women & Criminal *Justice*, 2, 83–105.

[19] Steffensmeier D. & Hebert C. (1999). Women and men policymakers: Does the judge's gender affect the sentencing of criminal defendants? *Social Forces*, 77(3), 1163–1196.

[20] Boyd C. L. (2016). Representation on the courts? The effects of trial judges’ sex and race. *Political Research Quarterly*, 69(4), 788–799.

[21] Peresie J. L. (2005). Female judges matter: Gender and collegial decisionmaking in the federal appellate courts. *The Yale Law Journal*, 114(7), 1759–1790.

[22] Allen D. W., & Wall D. E. (1993). Women state Supreme Court justices. *Judicature*, 77(3), 156–165.

[23] Anwar Shamena, Bayer Patrick, and Hjalmarsson Randi (2017). A jury of her peers: The impact of the first female jurors on criminal convictions. *The Economic Journal*, 129(618), 603–650.

[24] Albonetti C. A. (1992). Charge reduction: An analysis of prosecutorial discretion in burglary and robbery cases. *Journal of Quantitative Criminology*, 8(3), 317–333.

[25] Hartley Richard D., Maddan Sean, and Spohn Cassia C. (2007). Prosecutorial discretion: An examination of substantial assistance departures in federal crack‐cocaine and powder‐cocaine cases. *Justice Quarterly*, 24(3), 382–407.

[26] Kingsnorth R., Lopez J., Wentworth J., & Cummings D. (1998). Adult sexual assault: The role of racial/ethnic composition in prosecution and sentencing. *Journal of Criminal Justice*, 26(5), 359–371.

[27] LaFree G. D. (1980). The effect of sexual stratification by race on official reactions to rape. *American Sociological Review*, 45(5), 842–854. 7425435

[28] Mather L. M. (1979). *Plea Bargaining or Trial*? Lexington, MA: D.C. Heath.

[29] Paternoster R. (1984). Prosecutorial discretion in requesting the death penalty: A case of victim-based racial discrimination. *Law & Society Review*, 18(3), 437–478.

[30] Rehavi M. Marit, and Starr Sonja B. (2014). Racial disparity in federal criminal sentences. *Journal of Political Economy*,122(6), 1320–1354.

[31] Spohn C., & Holleran D. (2001). Prosecuting sexual assault: A Comparison of charging decisions in sexual assault cases involving strangers, acquaintances, and intimate partners.” *Justice Quarterly*, 18(3), 651–688.

[32] Spohn C., Gruhl J. & Welch S. (1987). The impact of the ethnicity and gender of defendants on the decision to reject or dismiss felony charges. *Criminology*, 25(1), 175–192.

[33] Wilmot K. A., & Spohn C. (2004). Prosecutorial discretion and real-offense sentencing: An analysis of relevant conduct under the federal sentencing guidelines. *Criminal Justice Policy Review*, 15(3), 324–343.

[34] Wooldredge J., & Thistlethwaite A. (2004). Bilevel disparities in court dispositions for intimate assault. *Criminology*, 42(2), 417–456 (2004).

[35] Metcalfe C. (2016). The role of courtroom workgroups in felony case dispositions: An analysis of workgroup familiarity and similarity. *Law & Society Review*, 50(3), 637–673.

[36] Lum K., & Ball P. (2015). Estimating undocumented homicides with two lists and list dependence. Human Rights Data Analysis Group. Available at: https://hrdag.org/wp-content/uploads/2013/01/2015-hrdag-estimating-undoc-homicides.pdf Accessed 2015-09-16.

[37] McCall M. (2005). Court decision making in police brutality cases, 1990–2000. *American Politics Research*, 33(1), 56–80.

[38] Shapiro R. Y., & Mahajan H. (1986). Gender differences in policy preferences: A summary of trends from the 1960s to the 1980s. *Public Opinion Quarterly*, 50(1), 42–61.

[39] Pratto F., Stallworth L.M., & Sidanius J. (1997). The gender gap: Differences in political attitudes and social dominance orientation. *British Journal of Social Psychology*, 36, 49–68. 10.1111/j.2044-8309.1997.tb01118.x 9114484

[40] Pratto F., Sidanius J. & Levin S. (2006). Social dominance theory and the dynamics of intergroup relations: Taking stock and looking forward.” *European Review of Social Psychology*, 17(1), 271–320.

[41] Smith T. W. (1984). The Polls: Gender and attitudes towards violence. *Public Opinion Quarterly*, 48(1), 384–396.

[42] Heaven P. C. L., & Bucci S. (2001). Right‐wing authoritarianism, social dominance orientation and personality: An analysis using the IPIP Measure. *European Journal of Personality*, 15(1), 49–56.

[43] Voyer D., & Voyer S.D. (2014). Gender differences in scholastic achievement: A meta-analysis. *Psychological Bulletin*, 140(4), 1174–1204. 10.1037/a0036620 24773502

[44] Fox R., & van Sickel R. (2000). Gender dynamics and judicial behavior in criminal trial courts: An exploratory study. *Justice System Journal*, 21(3), 261–280.

[45] Asp Petter. (2012). The prosecutor in Swedish law. *Crime and Justice*, 41(1), 141–165.

[46] Särskilda åklagarkammaren [Special Public Prosecution Office]. (2015). *Årsrapport 2014 [Annual report 2014]* Åklagarmyndigheten ÅM-A 2015/0287. Available at: https://www.aklagare.se/globalassets/dokument/rapporter/arsrapporter/2015_-_arsrapport_sarskilda_aklagarkammaren_2014.pdf. Accessed 2018-01-23.

[47] JO (Justitieombudsmannen/Parliamentary ombudsmen). 2018. *Inspektion av Åklagarmyndigheten, Sårskilda åklagarkammaren, den 5–7 december 2018*. Dnr. 2598–2018

[48] Loewen P. J., Koop R., Settle J., & Fowler J.H. (2014). A natural experiment in proposal power and electoral success. *American Journal of Political Science*, 58(1), 189–196.

[49] Polistidningen. 2018. Poliser som granskar poliser [Police who review police]. 5 December. Available at: https://polistidningen.se/2018/12/poliser-som-granskar-poliser/. Accessed 2020-01-15.

[50] SÅK, Särskilda åklagarkammaren [Special Public Prosecution Office]. (2020). Correspondence with Anders Jakobsson, Director of Public Prosecution dated 16 January 2020. Available in full in the online appendix.

[51] Dunning T. (2012). *Natural experiments in the social sciences*: *A design-based approach*. Boston, MA: Cambridge University Press.

[52] Crabtree C., & Fariss C.J. (2016). Stylized facts and experimentation. *Sociological Science*, 3, 910–914.

[53] Wooldridge J. M. (2010). *Econometric analysis of cross section and panel data*. Boston, MA: MIT Press.

[54] Abadie, A., Athey, S. Imbens, G. & Wooldridge, J. (2017). When should you adjust standard errors for clustering? Working paper. Available at https://economics.mit.edu/files/13927. Accessed 2018-01-23.

[55] Gerber A. S., & Green D. (2012). *Field experiments*: *design*, *analysis*, *and interpretation*. New York, NY: W. W. Norton and Company.

[56] Lin W., & Green D. (2015). Standard operating procedures: A safety net for pre-analysis plans. *PS*: *Political Science and Politics*, 49(3), 495–500.

[57] Macfarlane K. A. (2014). The danger of nonrandom case assignment: How the SDNY’s related cases’ rule has shaped the evolution of stop-and-frisk law. *Michigan Journal of Race and Law*, 19(2), 199–246.

[58] Adams R. B., & Ferreira D. (2009). Women in the boardroom and their impact on governance and performance. *Journal of Financial Economics*, 94, 291–309.

[59] Lindgren K.-O., Inkinen M. & Widmalm S. (2002). Who knows best what the people want: Women or men? A study of political representation in India. *Comparative Political Studies*, 42(1), 31–55.

